# NK cell dysfunction in severe COVID-19: TGF-β-induced downregulation of integrin beta-2 restricts NK cell cytotoxicity

**DOI:** 10.1038/s41392-022-00892-5

**Published:** 2022-01-31

**Authors:** Joana Barros-Martins, Reinhold Förster, Berislav Bošnjak

**Affiliations:** 1 Institute of Immunology, Hannover Medical School, 30625 Hannover, Germany; 2 Cluster of Excellence RESIST (EXC 2155), Hannover Medical School, 30625 Hannover, Germany; 3 German Centre for Infection Research (DZIF), Partner site Hannover, Hannover, Germany

**Keywords:** Infectious diseases, Innate immunity, Infectious diseases

In a recent study published in Nature, Witkowski et al. reported that in patients with severe forms of coronavirus disease 2019 (COVID-19) deranged tumor growth factor-beta (TGF-β) secretion counteracts interferon-alpha (IFN-α)-induced activation of NK cells by decreasing expression of transcription factor T-bet. Consequently, NK cells fail to upregulate the adhesion molecule integrin beta-2 (-ß2), which impedes their attachment to and killing of SARS-CoV-2 infected cells.^1^

Infection with severe acute respiratory syndrome coronavirus type-2 (SARS-CoV-2) results in divergent courses, ranging from asymptomatic infections to lethal COVID-19. Besides predisposing factors including various comorbidities, severe COVID-19 is also caused by misdirected, untimed, and/or hyper-activated immune responses. It is, therefore, crucial to characterize the role of individual leukocyte subsets in response to SARS-CoV-2 infection. NK cells, traditionally identified as CD3^−^CD56^+^ leukocytes, contribute to the early antiviral response by eliminating virus-infected cells. Their role in SARS-CoV-2 infection is still poorly understood. Although NK cells accumulate in the lungs of patients early after COVID-19 onset, in severe COVID-19 patients they were shown to have impaired antiviral activity.^2,3^

To determine the role of NK cells in protection against SARS-CoV-2, Witkowski et al. compared viral loads in COVID-19 patients with “normal” (> 40 NK/μl) or “low” (≤ 40 NK/μl) NK cell counts.^1^ They found that viral loads declined slower in patients with lower blood NK cell counts, suggesting that NK cells contribute to early infection control by eliminating SARS-CoV-2 infected cells. To validate their findings, the authors established an in vitro model in which NK cells were cultured with SARS-CoV-2 infected cell lines. In this model, NK cells from healthy donors dose-dependently killed infected cells, while NK cells from hospitalized COVID-19 patients were strongly impaired in mediating their protective function.

To validate those unexpected results, the authors investigated NK cell functions during the first 4 weeks of COVID-19 and of non-COVID-19-related flu-like illness (FLI). Compared to NK cells from healthy donors, NK cells in both patient groups had increased expression of the cytotoxic molecules perforin and granzyme B. However, NK cells from severe COVID-19 patients had significantly impaired cytotoxic activity and produced less IFN-γ and tumor necrosis factor-alpha (TNF-α) when compared to cells from ambulant COVID-19 or FLI patients. The authors further elegantly showed that impaired cytotoxicity is a consequence of inefficient attachment to the infected cells.

To understand the underlying molecular mechanisms, the authors evaluated the gene expression profiles of NK cells from COVID-19 patients with different disease manifestations by single-cell RNA sequencing (scRNA-seq). They reported that severe COVID-19 patients have higher numbers of proliferating NK cells and an increase of CD56^dim^ NK cells with a “terminally differentiated” profile, which is in line with previous flow cytometry analysis.^4^ While NK cells from all COVID-19 patients showed enrichment of IFN-α-regulated genes, only the cells from blood and lungs of severe COVID-19 patients were characterized by increased expression of genes driven by TGF-β, such as *EOMES* and *ITGAE*. This change in gene expression was evident upon comparing their expression signatures to scRNA-seq data of NK cells from healthy donors cultured in the presence of TGF-β, which revealed a negative regulation of *TBX21, STAT1*, and effector genes such as *PRF1* by this cytokine. Interestingly, TGF-β profoundly downregulated *ITGB2* that encodes for integrin-β2 (CD18), an adhesion molecule important for NK interaction with infected cells. NK cells isolated from FLI did not show a TGF-β-mediated gene expression profile.

To validate the scRNA-seq findings, the authors measured serum TGF-β levels in COVID-19 patients and found increased TGF-β concentration in the serum of hospitalized COVID-19 patients at the initial phase of the disease, which was not evident in serum of ambulant COVID-19 or FLI patients. Moreover, the authors observed increased *TGFB1* and *TGFB2* expression in SARS-CoV-2-infected lung resident cells such as type 1 alveolar epithelial cells, fibroblasts, myofibroblasts, and myeloid cells. Importantly, incubation of NK cells from healthy donors with serum from severe COVID-19 patients at early stages after symptom onset also let to impaired NK-mediated cytotoxicity that could be reversed by pre-treating with TGF-β blocking antibodies.

**Fig. 1:**
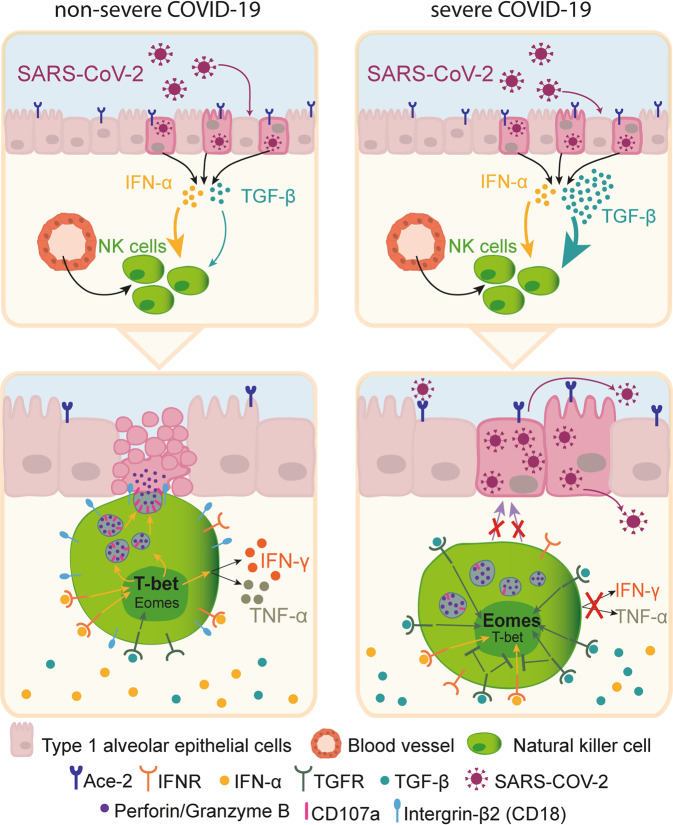
Production of tumor growth factor-beta (TGF-β) inhibits the antiviral functions of NK cells in response to SARS-CoV-2 infection. In severe but not in moderate COVID-19 patients, the high TGF-β concentration might be a result of SARS-CoV-2 infection (depicted) and/or co-morbidities. In severe COVID-19 patients, TGF-β is produced by various cell types, including type 1 alveolar epithelial cells (depicted), fibroblasts, myofibroblasts, and myeloid cells. TGF-β counteracts interferon-alpha (IFN-α)-induced activation of recruited natural killer (NK) cells, impedes T-bet activation, and skews the transcription activity towards Eomes. Consequently, these cells have lower expression of integrin-β2 (CD18), IFN-gamma (IFN-γ) and tumor necrosis factor alpha (TNF-α). Lack of integrin-β2 molecules precludes firm adhesion of NK cells to infected cells, preventing NK cell degranulation and killing of infected cells, thus leading to uncontrolled virus spread

Altogether, these data indicate that untimely TGF-β secretion in certain COVID-19 patients leads to downregulation of T-bet and, consequently, integrin-ß2 expression on NK cells, preventing them from successfully binding to and killing infected cells that eventually leads to increased viral loads and disease severity (Fig. 1). It will be interesting to see whether this mechanism also contributes to NK cell dysfunction in other diseases.

Of note, the early stage of COVID-19 is characterized by a cytokine storm and high levels of other cytokines such as type I interferons, IL-6, and IL-10 that have also been associated with different courses of disease progression. Liu et al. recently linked NK cell dysfunction in COVID-19 with high levels of IL-15 that induces their hyporesponsiveness.^5^ Another study also reported NK impairment in severe but not moderate COVID-19 patients but did not define the factors involved in NK cell suppression.^2^

Further studies investigating the interaction of TGF-β with other cytokines and their joint role on the function of NK cells and other immune cells are required to get better insights into the full list of factors leading to severe COVID-19. Moreover, it remains to be determined how increased TGF-β levels might affect functions of other cells, as this cytokine plays an important role in a myriad of biological processes reaching from immunosuppression to fibrosis induction. Furthermore, it is still not clear what causes the untimely peak of TGF-β that leads to NK cell impairment. Unfortunately, the described study does not mention whether severe patients suffered from any comorbidities that could have contributed to exacerbated TGF-β response and thus to the development of severe COVID-19. Nevertheless, implications of this important study could include the monitoring of plasma TGF-β levels in COVID-19 patients and test whether they could serve as an early indicator of disease progression as well as the possible use of TGF-β blockers in the treatment of COVID-19.
